# Recent Advances in Mycobacterial Research

**DOI:** 10.3390/jcm9082650

**Published:** 2020-08-14

**Authors:** Vishwanath Venketaraman

**Affiliations:** College of Osteopathic Medicine of the Pacific, Western University of Health Sciences, Pomona, CA 91766, USA; vvenketaraman@westernu.edu

Worldwide, tuberculosis (TB) remains the most frequent and important infectious disease that is responsible for causing significant morbidity and death [1,2]. One-third of the world’s population is infected with *Mycobacterium tuberculosis (Mtb)*, the etiologic agent of TB [1,2]. The World Health Organization estimates that eight to ten million new cases of TB occur annually worldwide, and the incidence of TB is currently increasing. In this context, TB is one of the top three (with malaria and HIV) leading causes of death from a single infectious agent, with approximately 1.6 million deaths attributable to TB annually. In particular, pulmonary TB, the most common form of TB, is a highly contagious and life-threatening infection [1,2].

*Mtb* infection originates in the lungs. Inhaled droplets containing Mtb are primarily engulfed by lung macrophages and dendritic cells that lead to the release of inflammatory mediators, which attracts other innate immune cells to the site of infection [2]. However, these innate responses are insufficient to control the progression of infection into symptomatic disease [2]. In most immuno-competent humans, the subsequent expansion of adaptive immunity, mediated mainly by CD4 and CD8 T-lymphocytes, results in the formation and evolution of granulomas where the infection is contained but not eradicated [2]. This condition where the infection continues to persist within the host at a sub-clinical level has been termed as latent TB (LTBI). The WHO has estimated that nearly a third of world’s population is harboring LTBI. However, individuals with LTBI can reactivate to symptomatic disease upon immune compromising conditions, such as HIV infection. 

Extra-pulmonary TB has become more common among individuals with advancing immunosuppression [3,4,5,6,7]. Central nervous system TB includes TB meningitis (TBM), intracranial tuberculomas, and spinal tuberculous arachnoiditis. TBM is the most common and severe form of extra-pulmonary TB and is associated with significant morbidity and mortality [8,9,10,11]. TBM, a medical emergency, is usually due to hematogenous dissemination of the tubercle bacillus and is still a major cause of serious illness in many parts of the world. TBM remains difficult to diagnose due to its broad, non-specific clinical spectrum. Improved outcome requires early recognition and treatment of these conditions [5,7,8,9,10,11]. Clinical features of TBM include the following: cerebral infarcts and mass lesions, fever for more than 7 days, headache and neck stiffness. Stroke occurs in 45% of patients with TBM both in early and later stage, mostly in basal ganglia region, and predicts poor outcome at 3 months [8,9,10,11]. Delay in the treatment of TBM results in either death or substantial neurological morbidity [8,9,10,11]. 

Nontuberculous mycobacteria (NTM) are a group of bacteria under the genus *Mycobacterium*, which includes *Mtb*, comprising more than 172 species with many unique virulence characteristics. NTM are found ubiquitously in the environment and present in water sources, soil, domestic and wild animals, and milk and food produce. NTM are opportunistic pathogens to animals and humans, including fish and poultry [12]. As opposed to TB caused by *M. tb* infection, NTM infection reporting is not mandatory; therefore, the incidence and prevalence of the different species of NTM are difficult to determine. However, the prevalence of NTM infection is growing in the United States, Europe, and other developed countries of the western world. The increased occurrences of NTM infections are associated with declining tuberculosis rates in areas of higher socioeconomic standards.

There is an urgent need to develop novel therapeutics and treatment strategies against mycobacterial infections. Host-directed therapy has been a potential target mechanism for effective clearance of infection. Host cell autophagy plays an essential role in antibacterial defense. The mammalian target of rapamycin (mTOR) has been negatively correlated with autophagy induction. Everolimus is an mTOR inhibitor that induces autophagy, but with higher water solubility. Therefore, targeting the mTOR pathway has the potential to develop novel and more effective combination drug therapies for TB. 

This Special Issue titled “Recent advances in mycobacterial research” includes a research article that demonstrates the beneficial effects of everolimus, both alone and in combination with current first-line antibiotics (isoniazid and pyrazinamide), in inhibiting the growth of *M. tb* inside in vitro human granulomas [13]. 

The role of atypical mycobacteria in causing pulmonary and extrapulmonary disease in the susceptible human population is also reviewed in this Special Issue with the main emphasis on *M. avium* and *M. abscessus*. In this review, the general characteristics of *M. avium* and *M. abscessus*, vulnerable human populations who are most at risk, pathogenesis, treatment, and prevention for infections caused by these two atypical mycobacteria are covered in detail [14].

Other important topics covered in this Special Issue include the following:

- The role of dendritic cells in regulating the immune responses against *Mtb* and HIV infections and strategies to improve the functions of dendritic cells in favor of the host.
- How upregulating glutathione by calcitriol along with diminishment in the levels of TGF-β can favor granulomatous responses against *Mtb* infection.
- Current knowledge on the pathogenesis, diagnosis and treatment of TB meningitis.
- How *Mycobacterium tuberculosis* accelerates HIV infection.
